# Community extension MSME's entrepreneurial activities in relation to poverty reduction

**DOI:** 10.3389/fsoc.2022.1038006

**Published:** 2022-11-28

**Authors:** Lislee Valle, Emily Costan, Felix Costan, Edralin General, Gerly Alcantara, Ronnel Victor Kilat, Ivy Batican, Gladies Mae Olivar, Denilin Avila

**Affiliations:** ^1^College of Education, Cebu Technological University-Danao Campus, Danao City, Philippines; ^2^College of Management and Entrepreneurship, Cebu Technological University-Danao Campus, Danao City, Philippines; ^3^College of Technology, Cebu Technological University-Danao Campus, Danao City, Philippines

**Keywords:** community extension, PLS-SEM (partial least squares - structural equation model), higher education institute, entrepreneurship, poverty reduction

## Abstract

**Introduction:**

Higher Education Institutions play a role in poverty reduction by implementing community extension programs focusing on capacity building and entrepreneurship training. Cebu Technological University programs offer these programs through various skills training to its targeted beneficiaries. This study aims to assess the community extension Micro-, Small and Medium-sized Enterprises (MSMEs) entrepreneurial activities concerning poverty reduction. The research participants are beneficiaries of community extension programs that primarily focus on capacity building and entrepreneurship training, and they eventually become entrepreneurs.

**Method:**

Using a cross-sectional survey, 172 valid responses were analyzed, with entrepreneurship education (EE), budgeting financial literacy (BFL), access to credit facilities (ACF), and entrepreneurial performance (EP) as predictors of poverty reduction (PR). Results from Partial Least Squares - Structural Equation Modeling (PLS-SEM) generate insights from the seven hypothesized paths of the proposed model.

**Results and discussion:**

Findings revealed that entrepreneurship education (β = 0.258, *p* < 0.05), budgeting financial literacy (β = 0.147, *p* < 0.05), and access to credit facilities (β = 0.541, *p* < 0.001) help reduce poverty. However, no significant relationship was found between entrepreneurial performance and poverty reduction (β = 0.132, *ns*) whose cause may be directly pointed to the pandemic's significant impact on MSME's entrepreneurial activities.

**Conclusion:**

This study confirms the importance of entrepreneurship education, budgeting, financial literacy, and credit access in promoting entrepreneurial success and reducing poverty.

## Introduction

Poverty is a multifaceted issue with numerous interconnections. Innovative ways of finding solutions are required to lessen poverty's effect on people, society, and the environment (Moniruzzaman and Day, 2020). In order to better understand poverty and develop strategies to combat it, various economic, social, structural, capacity, and learning techniques have been used to date (Sofo and Wicks, 2017). A rural community in the Philippines, like fishermen and farmers, remains the poorest primary sector in the Philippines (PSA, 2018). Due to the fact that Filipino farmers are still considered to be poor, it's necessary to give them an additional source of income (Yamagishi et al., 2021). Higher poverty rates are associated with poor rural subsistence on fishing and farming, illiteracy, unemployment, and larger family sizes (Cerio, 2019; Siphesihle and Lelethu, 2020).

Higher education institutions (HEIs) in the Philippines play an important role in reducing poverty (Yulo Loyzaga et al., 2022). Universities and colleges carry out a variety of entrepreneurship training through community extension programs that are offered to assist aspiring entrepreneurs. Community extension communicates and transfers knowledge and technology to specific sectors and target clientele, specifically those not enrolled in formal degree programs and course offerings (Palmén et al., 2020). The target beneficiaries can improve production, community, institution, and quality of life while enhancing HEI academic and research programs (CHED CMO No. 08-S. 2010). A unique ecosystem of collaboration of HEIs with communities, businesses, and industries facilitates the transfer of knowledge and technology in specific developmental sectors that directly impact the lives of individuals, families, and communities (Vermesan and Friess, 2022). In this case, extension is defined as the systematic transfer of technology, innovation, or information generated by HEIs and its partner to seek solutions to specific developmental concerns (Declaro-Ruedas et al., 2022). It is a purpose-specific, target-specific, and need-specific program of action utilizing the best available data, science, and evidence from various disciplines to inform systematic approaches to developmental solutions (Krasadakis, 2020). Research, innovation, and extension in Philippine higher education must work contextually and purposefully (Hirsu et al., 2021). Knowledge generation in HEIs should enable us to (a) deepen our understanding of ourselves as a people and as a nation and discover practical evidence- and science-based answers that can address real-world social, economic, and environmental challenges of families and communities (CHED CMO No. 52-S. 2016). Extension services are government-run programs that offer learning opportunities that support producers' technical ability and expertise (Ullah et al., 2021). These programs had been implemented to reach out to the local people for more significant development (ElMassah and Mohieldin, 2020). They influenced them to pursue a passion that maintains different aspects, especially employment, business, culture, norms, and values. Extension services are essential, and the best way to reach out to young people is through effective extension and advisory services (Ortiz-Crespo et al., 2021). Thus, it is possible to create extension programs that involve youths using participatory methodologies to provide input on program priorities (Ivanich et al., 2020).

In the Philippines, higher education institutions are thrust to strengthen university-community engagement through extension activities (Medina, 2018). For the aforementioned points to be taken seriously, it is important to keep in mind the following: (a) actions and decisions made in the form of a program have an impact on other people; (b) extension services planning is a coordinated effort that entails the identification, evaluation, and assessment of needs, issues, resources, priorities, and solutions; (c) while many other benefits, such as participant education, may result from the process, an extension services strategy is a matter that must be taken seriously. As a result, the function of extension programs continues to change. This technique facilitates an iterative, collaborative learning process where options are presented to users, who then modify them in response to local circumstances (Morrone, 2017; Villanueva et al., 2020).

The Cebu Technological University facilitates sustainable development in poor communities through extension services. CTU community extensions were made possible through the strong linkage between Cebu Technological University and the Local Government Unit. Ngaka and Zwane (2018) stressed that partnerships are indispensable in extension services. Similarly, partnerships can help reinforce, support, and even renovate individual partners, resulting in higher program quality, more well organized resource use, and better alignment of goals and programs (Weiss et al., 2010; Campos-Silva et al., 2021). As a result, several community extension services were successfully conducted with all support from the University Extension Services Office and the partner institution (Gutter et al., 2020; Antwi-Agyei and Stringer, 2021).

Most of CTU's extension services programs were designed to help the community to fight poverty (e.g., FEU community extension services 2020, Building Your Community Resources for Local Entrepreneurs 2021, and Creating Entrepreneurial Communities Conference 2022,). These programs were focused mainly on capacity building and entrepreneurship training. On the one hand, capacity building is the process whereby relevant stakeholders and organizations unleash, support, generate, acclimate and maintain capacity over time, usually to promise sustainable growth and improve the lives of the stakeholders (Jones et al., 2020; Loss et al., 2020; Casado-Asensio et al., 2022). It requires acquiring specific skills and developing opportunities to put the skills to prolific use (Issa et al., 2010; Harley et al., 2020; Ng et al., 2021). Full implementation of a well-designed capacity-building program ensures a sustainable extension service delivery system where extension workers can operate in the expected commercial economy (Issa, 2013; Costa and Andreaus, 2020). On the other hand, the goal of entrepreneurship programs for low-income self-employed people is to enhance their livelihood rather than promote cutting-edge innovation and business growth (Cho et al., 2016; George et al., 2021). Maziriri and Chivandi (2020) and Babajide et al. (2021) averred that entrepreneurship programs have variables that include entrepreneurship education, budget financial literacy, access to credit facilities, and entrepreneurial performance.

As an HEI, CTU's role in poverty reduction through entrepreneurship is carried out through its community extension programs that focus mainly on capacity building and entrepreneurship training, which offer various skills training to the beneficiaries. This paper evaluates the entrepreneurial activities of the community extension program by examining the variables that include entrepreneurship education, budget financial literacy, access to credit facilities, and entrepreneurial performance. Evaluating the effectiveness and sustainability of these programs that mainly assist the beneficiaries with becoming entrepreneurs impacts whether they should be forwarded, corrected, or terminated (Rahmat and Izudin, 2018).

The rest of the paper is organized into four sections: Section Literature review and hypothesis development presents the conceptual model and the hypotheses. Section Methods describes the methodological procedures. Section Data analysis and results reports the result of the PLS-SEM analysis. Section Discussions presents the discussion of the findings, while Section Recommendations provides concluding remarks, limitations, and some recommendations.

## Literature review and hypothesis development

### Entrepreneurship education and entrepreneurial performance

According to Manyaka-Boshielo (2019), entrepreneurship education (EE) is defined as ‘the skills and knowledge that individuals acquire through investment in schooling, on-the-job training, and other types of experience. While Mabenge et al. (2020) stated the ability of innovation to drive every firm activity, such as cost reduction, revenue growth, and aggressiveness, is referred to as entrepreneurial performance. The relationship between entrepreneurship education and entrepreneurial performance must be clarified. Van der Sluis et al. (2008) contend that investing in the education of imminent business visionaries results in greater entrepreneurial performance. The human capital idea affirms that previously acquired knowledge is crucial for academic execution. Coad et al. (2022) further point out that education enhances the performance of the entrepreneur in several areas, including business survival, firm development, and the association's entry into speculation. Based on the cases above, this theory is put forth:

H1: Entrepreneurship education positively and significantly impacts the entrepreneurial performance of MSMEs.

### Budgeting financial literacy and entrepreneurial performance

The capacity of managers to successfully manage money when making financial decisions is referred to as financial literacy (Marcolin and Abraham, 2006). According to Ripain et al. (2017), MSMEs play a crucial role in the economic development of many nations, and their performance and expansion have come to the attention of a number of stakeholders, including the government, policymakers, and financial institutions. The element of financial management is one of the most frequently mentioned success aspects of MSME, per Salikin et al. (2014). In Uasin Gishu County, Chepngetich (2016) observed a relationship between financial literacy and MSMEs' entrepreneurial success and discovered that financial literacy significantly impacted MSME performance. Further, Ibor et al. (2017) discovered that financial services had a favorable and significant impact on the functioning and expansion of MSMEs. Access to financial services is beneficial for MSMEs and other vulnerable and underprivileged businesses. According to Ratnawati (2020), financial inclusion has a direct and indirect impact on MSMEs' performance through the use of financial intermediation and capital access. As a result, we can speculate:

H2: Budgeting financial literacy positively affects the entrepreneurial performance of the community extension MSMEs

### Access to credit facilities and entrepreneurial performance

Kurgat et al. (2017) and Stubbs et al. (2021) defined a credit facility as an agreement with a bank or other credit institutions that enables a person or organization to borrow money when needed. Chege's (2014) and Maziriri and Chivandi (2020) investigation of the impact of credit facilities on the development of the 100 best MSMEs in Kenya found that access to credit facilities has a significant effect on the development of these enterprises. Therefore, the execution of an entrepreneurial venture relies on the entrepreneur's access to finance (Amouri et al., 2021). Enhancing the poor's access to financial services empowers them to develop beneficial resources and improve their profitability and potential for manageable jobs (Maziriri and Chivandi, 2020; Setiawan et al., 2020; Tisdell et al., 2020). When an MSME has access to credit facilities, it enhances the execution of its entrepreneurial endeavors (Madan, 2020). As such, the following hypothesis can be formulated

H3: Access to credit facilities positively affects the entrepreneurial performance of the community extension MSME.

### Entrepreneurial education and poverty reduction

For low-income individuals who want to start and grow their individual firms, entrepreneurship education can be helpful in delivering fundamental understanding, capabilities, and attitudes (Santos et al., 2019). Therefore, entrepreneurship provides reduced people a way out of poverty and an opportunity to enhance their society, create jobs, engage in self-employment, reduce crime, sustain their families, and realize other public welfare (Morris et al., 2020). Particularly during economic downturns (Lewis and Lee, 2020). Small-scale individual innovation and entrepreneurship have emerged as crucial elements in the fight to eradicate poverty following decades of large-scale state planning failure (Prideaux et al., 2020). Research in financial economics (Schwert, 2021) and applied economics and entrepreneurship. Karimi and Makreet (2020) recently affirmed the significance of entrepreneurship in the engagement of “bottom of the pyramid” consumers and the sensible decisions they may make regarding products and consumer welfare (Mehera and Ordonez-Ponce, 2021). As such, we can hypothesize:

H4: Entrepreneurship education positively affects poverty reduction

### Budgeting financial literacy and poverty reduction

Presented the method of developing a financial plan that projects future expenditures and income is known as budgeting (Warue and Wanjira, 2013; Moshashai et al., 2020; Srithongrung et al., 2021). On the one hand, Ayhan (2019), Sharif and Naghavi (2020), Pu et al. (2021) defined financial literacy as the information, services, and capability to direct increasingly multifaceted economic marketplaces. It is thought to give customers the power to make wise financial decisions. Additionally, Maziriri et al. (2018), Singla and Mallik (2021), Anshika and Singla (2022) emphasized that MSMEs' performance is significantly impacted by their lack of financial literacy regarding budgeting. Managers of rural MSMEs must become financially literate, specifically in budgeting (Gosal and Kamase, 2021). Thus, many Filipinos are suffering outward economic uncertainty from the deficiency of financial literacy, hindering their ability to survive during the pandemic. Therefore, we hypothesize that:

H5. Budgeting Financial Literacy positively affects poverty reduction

### Access to credit facilities and poverty reduction

Tasos et al. (2020) saw poverty as a universal fact that no one can deny; it was also recognized as the greatest catastrophic economic and social crisis mankind has encountered from its inception. This problem has made it difficult for most people to achieve their goals, such as receiving an education, earning money, starting businesses, or even finding a job. It takes a lot of effort to overcome poverty in many ways, depending on how an individual deals with all the situations. On the other hand, the governmental and private sectors have developed programs and advantages that could assist the Philippines in transcending poverty, such as expanding credit access. Implicating a credit facility, according to Chen et al. (2022), is a type of loan issued in the context of a business or corporate financing. It enables the borrowing company to borrow money over a longer time rather than reapplying for a loan each time it requires funds; in effect, a credit facility enables a company to take out an umbrella loan to generate capital over a longer period of time. Gichuki et al. (2014) stated that the high cost of payback, stringent collateral requirements, people's unwillingness to act as guarantors, expensive credit facility processing charges, and short repayment terms were prohibiting micro and small businesses from obtaining credit. As a result, it is recommended that financial institutions establish more flexible, affordable, and appealing terms for financing micro and small businesses. Water, electricity, and food are critical resources for regional social and economic development, according to Pan et al. (2022). They can be used as indicators or methods for measuring sustainable development goals to get finance, and the results can aid in the coordination of resource management and hence the reduction of poverty. The credit facilities will undoubtedly assist an individual in overcoming the challenge of poverty. Thus, we hypothesize that:

H6: Access to credit facilities positively affects poverty reduction

### Entrepreneurial performance and poverty reduction

Micro, Small, and Medium Enterprises (MSMEs) contribute significantly to income generation, employment creation, poverty reduction, and income inequality reduction (Marwa, 2014; Sriary and Nyoman, 2020). Small businesses, according to Matchaba-Hove et al. (2015), have an important role in improving development, progress, and vitality, as well as lowering unemployment and poverty. Similarly, Sarkar and Kumar, 2011; Egere et al., 2022 emphasized its importance in improving the socioeconomic conditions of the poor, creating job opportunities, allowing for greater exploitation of local raw materials and other resources, and boosting the country's economic progress. According to Sokoto and Abdullahi (2013), the potential of any organization to generate employment is critical in reducing the incidence of poverty among economic agents. Additionally, Yasa Kerti et al. (2013); Orji et al. (2022) conclude that improvements in MSME performance have a significant and fundamental impact on poverty reduction. The greater the change in MSME performance, the more significant the poverty reduction. Therefore, we can hypothesize,

H7: Entrepreneurial performance of the community extension MSME positively affects poverty reduction.

## Methods

### Research model

**Figure 1:**
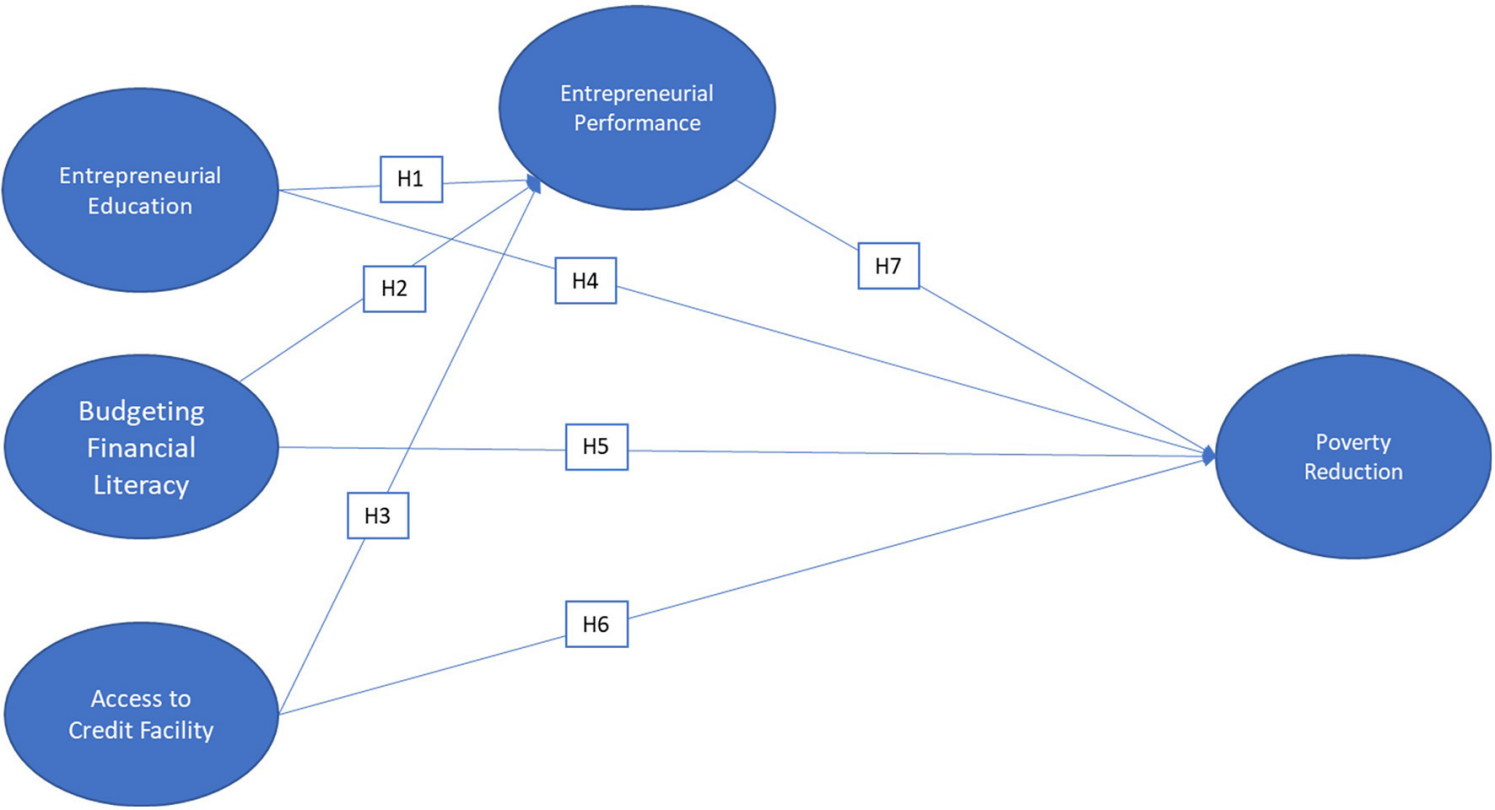
Model for entrepreneurial activities in relation to poverty reduction (adopted from Maziriri and Chivandi, 2020).

This paper adopted and modified the structural model of Maziriri and Chivandi (2020) of the key predictors that stimulate the entrepreneurial performance of small and medium enterprises (SMEs) and poverty. The aim of this study is to assess the community extension beneficiaries turned entrepreneurs and their entrepreneurial activities concerning poverty reduction during and after a crisis (e.g., health crisis, economic crisis due to calamities). Particularly, the effects of entrepreneurship education, budget financial literacy, access to credit facilities, and entrepreneurial performance in alleviating poverty. The structural equation model is shown in Figure 1.

### Sampling and data collection

The measurement items of each construct in this study were adopted from measures in prior works (see Appendix A). Entrepreneurial education (EE) has 13 measurement items, entrepreneurial performance (EP) has ten, poverty reduction has ten, access to credit facility has eight, and budgeting financial literacy has eight. The data were gathered through face-to-face questionnaire completion. The survey was distributed to around 200 participants for 4 weeks. There were 187 responses collected. The 15 responses had non-interactive responses and were subsequently removed. Of 187, only 172 were valid and used for the final analysis.

### Instruments

This study used a modified survey questionnaire to collect the necessary data. The questionnaire was divided into 3 sections. The first part includes questions such as the respondents' age, gender, special skills and community extension program. Second, the assessment of entrepreneurship education (Mwiya, 2014) and performance (Sariwulan et al., 2020), budgeting financial literacy (Siekei et al., 2013), access to credit facilities (Kurgat et al., 2017), and poverty reduction (Duclos and Tiberti, 2016) were gathered.

### Participants

A total of 172 community extension beneficiaries turned into micro-, small and medium sized entrepreneurs (56 males and 116 females). They were trained in different community extension programs; specifically, 46.02% attended Bread and Pastry, 17.44% had computer training, 15.12% detergent production, 14.53% was trained in both Coco water treated bottled milkfish in Spanish style with Rosemary Production and Coco water treated bottled milkfish sardines. Another 14.53% were trained in Virgin Coconut oil and Coconut water treated pork tocino production. Other training attended by at most 10% of the respondents were as follows; Kamias-based dishwashing liquid, rug making, t-shirt/mug printing, commercial cooking, sewing, cosmetology, and massage. The participants came from different municipalities in the Province of Cebu, Central Visayas Region. 6.40% of the participating entrepreneurs have below 40 hours of entrepreneurial education. 9.88% had 41–80 hours, 28.49% had 81–120 hours, while the majority of respondents, 54.65%, had 121 hours and above entrepreneurial education. All respondents volunteered to participate and were assured that their answers would be kept strictly confidential. The profile of the participants is reflected in Table 1.

**Table 1:** Profile of the respondents.

**Category**	***n***	**%**
**Age (in years)**		
Below 20	4	2.33
21–35	73	42.44
36–45	51	29.65
46–55 years old	26	15.12
56 and above	18	10.47
**Sex**		
Male	56	32.56
Female	116	67.44
**Num. of hours of entrepreneurial Educ**.		
Below 40 hours	11	6.4
41–80 hours	17	9.88
81–120 hours	49	28.49
121 and above	94	54.65
**Skills training attended**		
Bread and pastry	74	43.02
Computer	30	17.44
VCO based liquid handwash/detergent	26	15.12
Coco water treated bottled milkfish in Spanish style w/Rosemary	25	14.53
Coco water treated bottled milkfish sardines	25	14.53
Coconut water-treated pork tocino	25	14.53
Virgin coconut oil	25	14.53
Kamias based	18	10.47
Dishwashing liquid		
Rug making	18	10.47
T-shirt/mug printing	17	9.88
Commercial cooking	12	6.98
Sewing	11	6.4
Cosmetology	10	5.81
Massage	7	4.07

## Data analysis and results

This study utilized partial least-squared structural equation modeling (PLS-SEM) to determine the causal relationships between the investigated variables. PLS-SEM is a statistical technique that has become a potent method for examining correlations between variables, even in the presence of non-normality (Chinomona and Surujlal, 2012). Data were fed into Smart PLS software to ensure the internal consistency of the items within each section. Smart PLS statistical software estimates the parameters of the structural model and assesses the psychometric qualities of the measurement model. All survey questions required a forced response in order to guarantee that all responses were comprehensive and contained all necessary information. Furthermore, suspicious response patterns were deleted.

### Measurement model assessment

The PLS analysis allows parallel testing of the outer measurement model and the inner structural model and the presence of reflective and formative latent variables (Fornell and Bookstein, 1982). Since the proposed model in this study includes reflective measures, the first criterion in evaluating the model is to examine the measures' reliability and validity (Hair Jr et al., 2017). Based on the measurement model assessment result, all indicators were convergent and reliable, as shown in Table 2, where the factor loading for each item is greater than 0.70 (Henseler et al., 2009). Factor loadings less than 0.7 were removed (Chin, 1998). Nineteen item indicators (i.e., ACF2, ACF3, ACF4, ACF8, BFL1, BFL2, BFL3, EE1, EE2, EE3, EE4, EP1, EP3, EP9, PR1, PR2, PR3, PR4, and PR6) were removed after calculations through the SmartPLS algorithm until all the item indicators reached the threshold value of 0.70. There were 30 measurement indicators that remained for the final analysis. All measures for each construct were valid. With Average Variance Extracted (AVE) statistics greater than the threshold value of 0.5, all constructs have appropriate convergent validity (Fornell and Larcker, 1981), ranging from 0.609 to 0.768. Furthermore, the measurement items were all reliable, with all the constructs garnered above the Cronbach's alpha (α) threshold value of 0.60, which is considered of acceptable reliability and an acceptable index (Nunnally, 1994; Ursachi et al., 2015) and composite reliability (CR) threshold value of 0.70 (Hair Jr et al., 2017). The Cronbach's alpha ranges from 0.893 to 0.927, while the CR values range from 0.895 to 0.932. These results indicate high-reliability values. Table 2 provides a summary of the measurement model results.

**Table 2:** Measurement model assessment results.

**Items**	**Loadings**	**AVE**	**Cronbach α**	**CR**
ACF1	0.806	0.768	0.898	0.9
ACF5	0.893			
ACF6	0.91			
ACF7	0.893			
BFL4	0.778	0.723	0.906	0.926
BFL5	0.889			
BFL6	0.837			
BFL7	0.87			
BFL8	0.872			
EE5	0.813	0.633	0.927	0.932
EE6	0.793			
EE7	0.707			
EE8	0.832			
EE9	0.855			
EE10	0.722			
EE11	0.798			
EE12	0.799			
EE13	0.828			
EP2	0.769	0.609	0.893	0.895
EP4	0.816			
EP5	0.782			
EP6	0.793			
EP7	0.767			
EP8	0.787			
EP10	0.749			
PR5	0.767	0.704	0.894	0.896
PR7	0.854			
PR8	0.848			
PR9	0.857			
PR10	0.866			

α, Cronbach's alpha; CR, composite reliability; AVE, average variance extracted; ACF, access to credit facility; BLT, budgeting financial literacy; EE, entrepreneurial education; EP, entrepreneurial performance; PR, poverty reduction.

The correlations of the measures of potential overlapping variables are used to assess the degree to which the measurement items measure distinctively among constructs (Hair Jr et al., 2014). The square root of AVE was calculated to ensure discriminant validity. The AVE of the constructs was found to support discriminant validity because it is greater than the squared correlation of each latent variable (Fornell and Larcker, 1981). Table 3 bolds the square roots of the AVE, while non-bolded values represent the intercorrelation value between constructs. All off-diagonal values are less than the square roots of AVE, indicating that Fornell and Larker's condition is satisfied. Overall, the measurement model's reliability and validity tests were met. All items used in this study to measure constructs are valid and fit to estimate parameters in the structural model.

**Table 3:** Fornell and Larcker results.

	**ACF**	**BFL**	**EE**	**EP**	**PR**
ACF	**0.876**				
BFL	0.159	**0.85**			
EE	0.683	0.075	**0.795**		
EP	0.772	0.243	0.633	**0.781**	
PR	0.842	0.246	0.7	0.748	**0.839**

Square root of AVE is shown on the diagonal of the matrix in bold; inter-construct correlation is shown off the diagonal.

In Table 3, the research model fitness demonstrates an acceptable fit with a Standardized Root Mean Square Residual (SRMR) value of 0.077 and a common acceptable fit value of 0.08. The Normed Fit Index (NFI) value is 0.696, reflecting a moderate acceptable value with the threshold of NFI < 0.90. The NFI generates values ranging from 0 to 1. The closer the NFI is to one, the better the fit. In general, NFI values greater than 0.9 imply an excellent fit.

### Structural model

**Figure 2:**
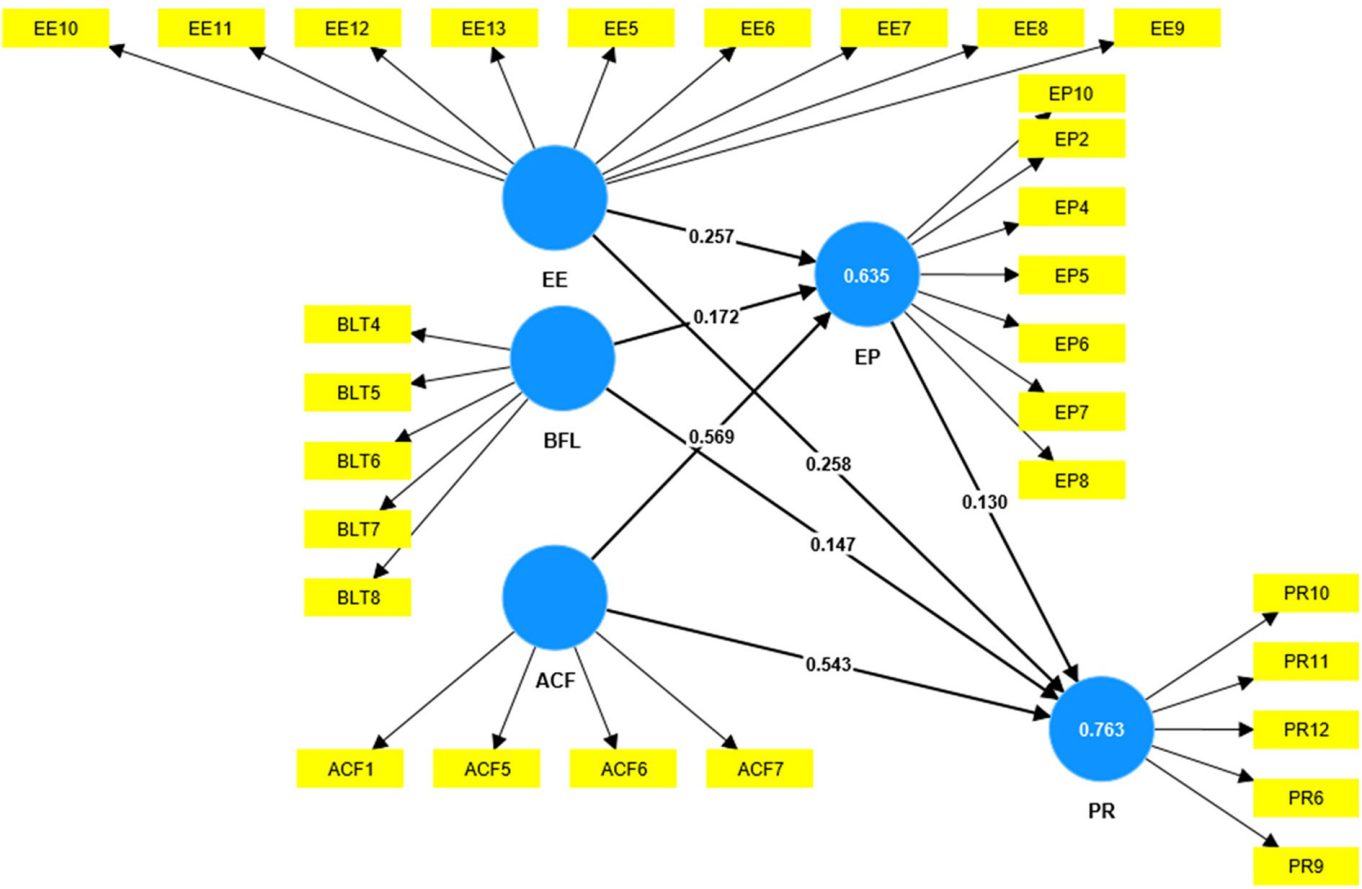
The final result.

The influence of the independent variables on the dependent variable is tested using a structural model (Hair Jr et al., 2014). When using PLS-SEM, the three main factors used to assess the structural model are the strength of the path coefficients, R^2^ values (prediction power), and f2 (effect size) (Hair Jr et al., 2017). The path coefficients of the structural model indicate that the six hypotheses are supported (H1, H2, H3, H4, H5, H6), and only one is not supported (H7). The results are summarized in Table 4 (Figure 2). The acceptable values of 0.75, 0.50, and 0.25 correspond to significant, moderate, and modest levels of prediction accuracy, respectively (Henseler et al., 2009; Hair Jr et al., 2014). The coefficient of determination (*R*^2^) in this study provides the predictive accuracy of the structural model, as indicated in Figure 2. PR is explained to have the highest variance with a value of 0.763 (76%), while EP has a value of 0.635 (63.5%). Thus, the *R*^2^ criterion is met, and the predictive ability of the structured model is considered moderately high.

**Table 4:** Path coefficient results.

	**β**	***t* values**	***p*-values**	**Decision**
ACF -> EP	0.573	5.588	0.000***	Supported
ACF -> PR	0.541	6.455	0.000***	Supported
BFL -> EP	0.171	1.992	0.046**	Supported
BFL -> PR	0.147	1.964	0.049**	Supported
EE -> EP	0.253	2.599	0.009**	Supported
EE -> PR	0.258	3.176	0.002**	Supported
EP -> PR	0.132	1.386	0.166^ns^	Not Supported

*** *p* < 0.001;

** *p* < 0.05; *ns*, not significant.

The effect sizes (*f*^2^) were estimated using the SmartPLS algorithm, indicative of a minor, medium, or substantial effect on the link between exogenous and endogenous constructs with *f*^2^ values of 0.02, 0.15, and 0.35, respectively (Hair Jr et al., 2017). A value less than 0.02 indicates that exogenous constructs do not affect endogenous constructs. The *f*^2^ results show that ACF substantially affects EP (*f*^2^ = 0.444), and ACF substantially affects PR (*f*^2^ = 0.434). Furthermore, EP has a small effect on PR (*f*^2^ = 0.026). These results, as indicated in Table 5, are consistent with the other findings in this study.

**Table 5:** Effect size results.

	**ACF**	**BFL**	**EE**	**EP**	**PR**
ACF				0.444	0.434
BFL				0.076	0.08
EE				0.093	0.132
EP					0.026
PR					

## Discussion

This section presents the salient features of the PLS-SEM analysis and how these results can be applied to the current discussions of Community Extension MSME's entrepreneurial activities in relation to poverty reduction. The flow followed the discussions from the antecedent variables toward the model and how it affects entrepreneurial performance and poverty reduction.

It can be seen in Table 4 that Entrepreneurial performance directly impacts entrepreneurship education (β = 0.253), budgeting financial literacy (β = 0.171), and Access to credit facilities (β = 0.573). The results of the hypothesis testing are congruent with the findings of Green et al. (2006). For example, if the expansion of the business is due to the application of transferred technology, it helps to seek better business opportunities. Further, if the entrepreneurial performance encourages business start-ups, then the level of budgeting skill is relevant to the business ventures. Similarly, if the entrepreneurial performance creates something to take advantage of the creative needs, it can improve the level of income in the business. It can be noted that entrepreneurial performance directly impacts entrepreneurship education, budgeting financial literacy, and access to credit facilities. Thus, if entrepreneurial performance can control the business and motivate the businessmen and entrepreneurs who show low interest in the business, they would tend to pursue business because they believe it can improve the standard of living and have a potential unlimited income.

The PLS-SEM analysis discloses that entrepreneurship education (β = 0.258), budgeting finance literacy (β = 0.147) and access to credit facilities (β = 0.541) directly impacts poverty reduction. Further, this collaborates with the study of Sokoto and Abdullahi (2013), Pan et al. (2022). For instance, if food scarcity has been reduced, it empowers me to strive for a better standard of living. Moreover, if the family members are sent to school, they will acquire skills in profit planning, business financing, and cash flow management. Additionally, if the family can buy additional appliances, then access to credit facilities is empowered to own property.

The path coefficient reflected in Table 4 shows that the EP to PR (β = 0.132) were not supported. Since the advent of the coronavirus, COVID-19 has generated one of the most urgent crises at the global level in recent times; with the steepest downgrades in economic growth among all global recessions, there is no significant relationship between entrepreneurial performance and poverty reduction (Parnell et al., 2020; Ratten, 2020; Crupi et al., 2022). It has a negative impact on aspiring entrepreneurs, particularly those from developing nations where government help is restricted (Nasar et al., 2019). Authorities' lockdowns and movement control orders are the most significant elements influencing entrepreneurial activity (Nasar et al., 2019; Ionescu-Somers and Tarnawa, 2020; Perveen et al., 2022). The epidemic has also resulted in low demand and market stagnation, making it more difficult for entrepreneurs to continue their start-up projects. The business environment has heightened the dread of failure, with maximal risks of ceasing or reducing entrepreneurial activities (Nasar et al., 2019). The fear factor was highlighted as an essential indication restricting potential and embryonic entrepreneurs' entrepreneurial activities (Li, 2011; Morgan and Sisak, 2016). Because the economic implications would continue longer, the entrepreneurial activity would decline regardless of whether they were located in a developed or developing country (Nasar et al., 2022). Entrepreneurs have suffered greatly due to COVID-19's social distancing rules and other altered corporate operating processes (Nasar et al., 2022). Entrepreneurs encounter complex challenges to preserve their standing because they use opportunities to address problems and develop goods that help society (Williams et al., 2017).

## Recommendations

Based on the research findings, the following claims are made: Credit finance promotes development by allowing MSMEs to engage in profitable ventures that frequently necessitate large capital investments. As a result, financial lending institutions should consider lowering collateral requirements to make it easier for MSMEs to access and promote their activities. Furthermore, in order to make good financial decisions, MSMEs must acquire financial literacy skills, specifically budgeting financial literacy. On the other hand, entrepreneurial education strives to provide expertise, entrepreneurship skills, and inspiration among entrepreneurs; consequently, aspiring entrepreneurs should take. Also, MSME entrepreneurs should be given education and assistance in times of calamities, natural disasters, and global pandemics since they are vital in the economy's recovery. Finally, MSMEs should be given proper attention by allocating additional resources to the sector, particularly in the aftermath of the COVID-19 outbreak.

## Conclusion and limitation of the study

The objective of this study was to look into the effects of entrepreneurship education, budgeting, and financial literacy, and access to credit on entrepreneurial performance and poverty reduction. The study confirms the importance of entrepreneurship education, budgeting, financial literacy, and credit access in promoting entrepreneurial success and reducing poverty. Entrepreneurial performance was found to have a greater impact on poverty reduction than entrepreneurship education and access to credit facilities. A strong correlation was also established between budgeting financial literacy and entrepreneurial performance. Except for hypothesis 7, which was positive but insignificant, the findings confirm all of the stated assumptions. The implications of the findings and future research goals were highlighted. However, this study has some limitations that must be considered. This used the cross-sectional research design; specifically, the sample population of the community extension beneficiaries turned into micro-small-medium entrepreneurs and the data was collected during the start of the economic recovery caused by the COVID-19 pandemic. Overall, this study will contribute to the existing knowledge in entrepreneurship and small business management. In academia, this study context is currently under-researched and under-appreciated.

## Data availability statement

The raw data supporting the conclusions of this article will be made available by the authors, without undue reservation.

## Ethics statement

Ethical review and approval was not required for the study on human participants in accordance with the local legislation and institutional requirements. The patients/participants provided their written informed consent to participate in this study.

## Author contributions

All authors listed have made a substantial, direct, and intellectual contribution to the work and approved it for publication.

## Funding

This paper was funded by Cebu Technological University, Cebu City, Philippines.

## Conflict of interest

The authors declare that the research was conducted in the absence of any commercial or financial relationships that could be construed as a potential conflict of interest.

## Publisher's note

All claims expressed in this article are solely those of the authors and do not necessarily represent those of their affiliated organizations, or those of the publisher, the editors and the reviewers. Any product that may be evaluated in this article, or claim that may be made by its manufacturer, is not guaranteed or endorsed by the publisher.

## References

[B1] Amouri A. Festa G. Shams S. R. Sakka G. Rossi M. (2021). Technological propensity, financial constraints, and entrepreneurial limits in young entrepreneurs' social business enterprises: the tunisian experience. Technol. Forecast. Soc. Chang. 173, 121126. 10.1016/j.techfore.2021.121126

[B2] Anshika A. Singla A. (2022). Financial literacy of entrepreneurs: a systematic review. Manag. Financ. 48, 1352–1371. 10.1108/MF-06-2021-0260

[B3] Antwi-Agyei P. Stringer L. C. (2021). Improving the effectiveness of agricultural extension services in supporting farmers to adapt to climate change: insights from northeastern Ghana. Clim. Risk Manag. 32, 100304. 10.1016/j.crm.2021.100304

[B4] Ayhan B. (2019). Constituting financialized subjectivities: cultural political economy of financial literacy in Turkey. Turk. Stud. 20, 680–707. 10.1080/14683849.2018.1520103

[B5] Babajide A. Osabuohien E. Tunji-Olayeni P. Falola H. Amodu L. Olokoyo F.. (2021). Financial literacy, financial capabilities, and sustainable business model practice among small business owners in Nigeria. J. Sustain. Financ. Invest. 1–23. 10.1080/20430795.2021.1962663

[B6] Campos-Silva J. V. Peres C. A. Hawes J. E. Haugaasen T. Freitas C. T. Ladle R. J. Lopes P. F. (2021). Sustainable-use protected areas catalyze enhanced livelihoods in rural Amazonia. Proc. Natl. Acad. Sci. 118, e2105480118. 10.1073/pnas.2105480118 34580218 PMC8501803

[B7] Casado-Asensio J. Blaquier D. Sedemund J. (2022). Strengthening Capacity for Climate Action in Developing Countries: Overview and Recommendations. Retrieved from: https://www.oecd-ilibrary.org/development/strengthening-capacity-for-climate-action-in-developing-countries_0481c16a-en

[B8] Cerio C. T. (2019). Revisiting the sociological theories of poverty: conceptualising a framework for rural poverty in the Philippines. Trans. J. Rural Res. 1, 33–52. 10.5281/zenodo.2612927

[B9] Chege G. S. (2014). The effect of access to credit facilities on the growth of top 100 small and medium enterprises in Kenya (Doctoral dissertation). University of Nairobi, Nairobi, Kenya.

[B10] Chen H. Ni D. Zhu S. Ying Y. Shen M. (2022). Does the National Credit Demonstration Policy Affect Urban Green Economy Efficiency? Evidence from the Yangtze River Delta Region of China. Int. J. Environ. Res. Public Health. 19, 9926. 10.3390/ijerph19169926 36011553 PMC9408644

[B11] Chepngetich P. (2016). Effect of financial literacy and performance SMEs. Evidence from Kenya. *Am. Res. J*. 5:26–35. 10.5281/zenodo.3441820

[B12] Chin W. W. (1998). Commentary: Issues and opinion on structural equation modeling. MIS Quart. 2, vii-xvi. Retrieved from: https://www.jstor.org/stable/249674

[B13] Chinomona R. Surujlal J. (2012). The influence of student internship work experience on their self-improvement and professionalism in Sport Management: sport management. Afr. J. Phys. Health Educ. Recreat. Dance 18, 885–899. Retrieved from: https://journals.co.za/doi/epdf/10.10520/EJC128369

[B14] Cho Y. Robalino D. Watson S. (2016). Supporting self-employment and small-scale entrepreneurship: potential programs to improve livelihoods for vulnerable workers. IZA J. Labour Policy. 5, 1–26. 10.1186/s40173-016-0060-2

[B15] Coad A. Harasztosi P. Pál R. Teruel M. (2022). Policy instruments for high-growth enterprises. Quest. Entrepr. State 273–98. 10.1007/978-3-030-94273-1_15

[B16] Costa E. Andreaus M. (2020). Social impact and performance measurement systems in an Italian social enterprise: a participatory action research project. J. Public Budget. Acc. Financ. Manag. 33, 289–313. 10.1108/JPBAFM-02-2020-0012

[B17] Crupi A. Liu S. Liu W. (2022). The top-down pattern of social innovation and social entrepreneurship. Bricolage and agility in response to COVID-19: cases from China. RandD Manag 52, 313–330. 10.1111/radm.12499

[B18] Declaro-Ruedas M. Y. A. Bais L. S. Munar J. L. (2022). Navigating new normal extension management strategies in occidental Mindoro state college. Soc. Sci. 11, 78–84. 10.11648/j.ss.20221102.14

[B19] Duclos J. Y. Tiberti L. (2016). Multidimensional Poverty Indices: A Critical Assessment. 10.2139/ssrn.2718374

[B20] Egere O. M. Maas G. Jones P. (2022). A critical analysis of the Nigerian entrepreneurial ecosystem on transformational entrepreneurship. J. Small Bus. Manag. 1–32. 10.1080/00472778.2022.2123109

[B21] ElMassah S. Mohieldin M. (2020). Digital transformation and localizing the sustainable development goals (SDGs). Ecol. Econ. 169, 106490. 10.1016/j.ecolecon.2019.106490

[B22] Fornell C. Bookstein F. L. (1982). Two structural equation models: LISREL and PLS applied to consumer exit-voice theory. J. Mark. Res. 19, 440–452. 10.1177/002224378201900406

[B23] Fornell C. Larcker D. F. (1981). Evaluating structural equation models with unobservable variables and measurement error. J. Mark. Res. 18, 39–50. 10.1177/002224378101800104

[B24] George T. O. Oladosun M. Oyesomi K. Orbih M. U. Nwokeoma N. Iruonagbe C. Lawal-Solarin E. (2021). Usefulness and expectations on skills development and entrepreneurship among women of low socioeconomic status in Ogun State, Nigeria. Afr. J. Reprod. Health 25, 171–187. Retrieved from: https://ajrh.info/index.php/ajrh/article/view/2998 10.29063/ajrh2021/v25i5s.16 37585781

[B25] Gichuki J. A. W. Njeru A. Tirimba O. I. (2014). Challenges facing micro and small enterprises in accessing credit facilities in Kangemi Harambee market in Nairobi City County, Kenya. Int J Scient. Public. 4, 1–25. Available online at: http://www.ijsrp.org/research-paper-1214.php?rp=P363434

[B26] Gosal B. Kamase R. (2021). Identification of financial literacy level–case study of small business owner or manager in gowa regency. J Manag Er 1, 87–98. Retrieved from: http://ojs.feb.uajm.ac.id/index.php/jmer/article/view/236/123

[B27] Green C. J. Kirkpatrick C. H. Murinde V. (2006). Finance for small enterprise growth and poverty reduction in developing countries. J. Int. Develop. 18, 1017–1030. 10.1002/jid.1334

[B28] Gutter M. S. O'Neal L. J. Riportella R. Sugarwala L. Mathias J. Vilaro M. J. Rhyne R. (2020). Promoting community health collaboration between CTSA programs and cooperative extension to advance rural health equity: insights from a national Un-Meeting. J. Clin. Transl. Sci. 4, 377–383. 10.1017/cts.2020.13 33244425 PMC7681129

[B29] Hair Jr J. F. Sarstedt M. Hopkins L. Kuppelwieser V. G. (2014). Partial least squares structural equation modeling (PLS-SEM): an emerging tool in business research. Eur. Bus. Rev. 26, 06–121 10.1108/EBR-10-2013-0128

[B30] Hair Jr J. F. Sarstedt M. Ringle C. M. Gudergan S. P. (2017). Advanced Issues in Partial Least Squares Structural Equation Modeling. Thousand Oaks, CA: SAGE publications.

[B31] Harley D. Grome S. Kim S. H. McLendon T. Hunn V. Canfield J. Wells A. (2020). Perceptions of success and self-sustainability among women participating in an entrepreneurial skills development and empowerment program through photovoice. J. Ethnic Cult. Diver. Soc. Work 29, 377–395. 10.1080/15313204.2017.1344900

[B32] Henseler J. Ringle C. M. Sinkovics R. R. (2009). The use of partial least squares path modeling in international marketing, in New Challenges to International Marketing. Bingley: Emerald Group Publishing Limited. 20, 277–319. 10.1108/S1474-7979(2009)000002001424918859

[B33] Hirsu L. Quezada-Reyes Z. Hashemi L. (2021). Moving SDG5 forward: women's public engagement activities in higher education. High. Educ. 81, 51–67. 10.1007/s10734-020-00597-0

[B34] Ibor B. I. Offiong A. I. Mendie E. S. (2017). Financial inclusion and performance of micro, small and medium scale enterprises in Nigeria. Int. J. Res. Granthaalayah. 5, 104–122. 10.5281/zenodo.439557

[B35] Ionescu-Somers A. Tarnawa A. (Eds.). (2020). Diagnosing COVID-19 Impacts on Entrepreneurship: Exploring Policy Remedies for Recovery. London: Global Entrepreneurship Research Association, London Business School.

[B36] Issa F. O. (2013). Building the capacity of agricultural extension personnel for effective implementation of agricultural transformation agenda in Nigeria. J. Agri. Exten. 17, 78–88. 10.4314/jae.v17i1.8

[B37] Issa T. Chang V. Issa T. (2010). Sustainable business strategies and PESTEL framework. GSTF Int. J. Comput. 1, 73–80. 10.5176/2010-2283_1.1.13

[B38] Ivanich J. D. Mousseau A. C. Walls M. Whitbeck L. Whitesell N. R. (2020). Pathways of adaptation: two case studies with one evidence-based substance use prevention program tailored for indigenous youth. Prev. Sci. 21, 43–53. 10.1007/s11121-018-0914-5 29876790 PMC6774906

[B39] Jones G. J. Edwards M. B. Bocarro J. N. Svensson P. G. Misener K. (2020). A community capacity building approach to sport-based youth development. Sport Manag. Rev. 23, 563–575. 10.1016/j.smr.2019.09.001

[B40] Karimi S. Makreet A. S. (2020). The role of personal values in forming students' entrepreneurial intentions in developing countries. Front. Psychol. 11, 525844. 10.3389/fpsyg.2020.525844 33329168 PMC7710526

[B41] Krasadakis G. (2020). A framework for innovation, in The Innovation Mode (Cham: Springer, Cham), pp. 59–92. 10.1007/978-3-030-45139-4_4

[B42] Kurgat F. Owembi K. O. Omwono G. A. (2017). Impact of access to credit facilities on youth economic development: a case of Mwanzo youths in Uasin Gishu County, Kenya. Int. J. Res. Bus. Stud. Manag. 4, 24–36. 10.22259/ijrbsm.0401004

[B43] Lewis M. Lee A. J. (2020). Affording health during the COVID-19 pandemic and associated economic downturn. Aust. NZ. J. Public Health. 44, 519–520. 10.1111/1753-6405.13045 33047419 PMC7675258

[B44] Li Y. (2011). Emotions and new venture judgment in China. Asia Pac. J. Manag. 28, 277–229. 10.1007/s10490-009-9145-4

[B45] Loss J. Brew-Sam N. Metz B. Strobl H. Sauter A. Tittlbach S. (2020). Capacity building in community stakeholder groups for increasing physical activity: results of a qualitative study in two German communities. Int. J. Environ. Res. Public Health 17, 2306. 10.3390/ijerph17072306 32235419 PMC7177804

[B46] Mabenge B. K. Ngorora-Madzimure G. P. K. Makanyeza C. (2020). Dimensions of innovation and their effects on the performance of small and medium enterprises: The moderating role of firm's age and size. J. Small. Entrepreneurship. 34, 1–25. 10.1080/08276331.2020.1725727

[B47] Madan N. (2020). A Review of Access to Finance by Micro, Small and Medium Enterprises and Digital Financial Services in Selected Asia-Pacific Least Developed Countries. Retrieved from: https://www.unescap.org/sites/default/files/publications/WP_20_03%20MSMEs%20in%20LDCs.pdf

[B48] Manyaka-Boshielo S. J. (2019). Towards entrepreneurship education: Empowering township members to take ownership of the township economy. HTS Theol. Stud. 75, a5166. 10.4102/hts.v75i1.5166

[B49] Marcolin S. Abraham A. (2006). Financial Literacy Research: Current Literature and Future Opportunities. In 3rd International Conference of Contemporary Business 2006. Retrieved from: https://ro.uow.edu.au/cgi/viewcontent.cgi?article=1233&context=commpapers

[B50] Marwa N. (2014). Micro, Small and Medium Enterprises' External Financing Challenges: The Role of Formal Financial Institutions and Development Finance Intervention in Tanzania. 10.7763/IJTEF.2014.V5.376

[B51] Matchaba-Hove T. Farrington S. Sharp G. (2015). The entrepreneurial orientation-Performance relationship: A South African small business perspective. The Southern African Journal of Entrepreneurship and Small Business Management, 7(1), pp.36–68. 10.4102/sajesbm.v7i1.6

[B52] Maziriri E. T. Chivandi A. (2020). Modelling key predictors that stimulate the entrepreneurial performance of small and medium-sized enterprises (SMEs) and poverty reduction: Perspectives from SME managers in an emerging economy. Acta Commercii 20, 1–15. 10.4102/ac.v20i1.773

[B53] Maziriri E. T. Mapuranga M. Madinga N. W. (2018). Self-service banking and financial literacy as prognosticators of business performance among rural small and medium-sized enterprises in Zimbabwe. South. Afr. J. Entrep. Small Bus. Manag. 10, 1–10. 10.4102/sajesbm.v10i1.180

[B54] Medina M. A. P. (2018). A community extension framework for Philippine higher education institutions: A model developed from small-scale climate change adaptation projects of Central Mindanao University. World Sci. News 105, 204–211. Retrieve from: http://www.worldscientificnews.com/wp-content/uploads/2018/07/WSN-105-2018-204-211.pdf

[B55] Mehera A. Ordonez-Ponce E. (2021). Social and economic value creation by Bendigo Bank and Stockland Property Group: Application of shared value business model. Bus. Soc. Rev. 126, 69–99. 10.1111/basr.12224

[B56] Moniruzzaman M. Day R. (2020). Gendered energy poverty and energy justice in rural Bangladesh. Energy Policy 144, 111554. 10.1016/j.enpol.2020.111554

[B57] Morgan J. Sisak D. (2016). Aspiring to succeed: a model of entrepreneurship and fear of failure. J. Bus. Vent. 31, 1–21 10.1016/j.jbusvent.2015.09.002

[B58] Morris M. H. Soleimanof S. White R. J. (2020). Retirement of entrepreneurs: Implications for entrepreneurial exit. J. Small. Bus. Manag. 58, 1089–1120. 10.1111/jsbm.12476

[B59] Morrone V. (2017). Outreach to support rural innovation, in Agricultural Systems (Academic Press), 407–439. 10.1016/B978-0-12-802070-8.00012-8

[B60] Moshashai D. Leber A. M. Savage J. D. (2020). Saudi Arabia plans for its economic future: vision 2030, the national transformation plan and Saudi fiscal reform. Br. J. Mid. East. Stud. 47, 381–401. 10.1080/13530194.2018.1500269

[B61] Mwiya B. M. K. (2014). The Impact of Entrepreneurship Education on the Relationships Between Institutional and Individual Factors and Entrepreneurial Intention of University graduates : Evidence from Zambia.

[B62] Nasar A. Akram M. Safdar M. R. Akbar M. S. (2022). A qualitative assessment of entrepreneurship amidst COVID-19 pandemic in Pakistan. Asia Pacific Manag. Rev. 27, 182–189. 10.1016/j.apmrv.2021.08.001

[B63] Nasar A. Kamarudin S. Rizal A. M. Ngoc V. T. B. Shoaib S. M. (2019). Short-term and long-term entrepreneurial intention comparison between Pakistan and Vietnam. Sustainability 11, 6529 10.3390/su11236529

[B64] Ng P. M. Chan J. K. Wut T. M. Lo M. F. Szeto I. (2021). What makes better career opportunities for young graduates? Examining acquired employability skills in higher education institutions. Educ. Train. 10.1108/ET-08-2020-0231

[B65] Ngaka M. J. Zwane E. M. (2018). The role of partnerships in agricultural extension service delivery: a study conducted in provincial departments of agriculture in South Africa. South African Journal of Agricultural Extension 46, 14–25. 10.17159/2413-3221/2018/v46n1a3992018

[B66] Nunnally J. C. (1994). Psychometric Theory 3rd Edn. New York, NY: Tata McGraw-hill education.

[B67] Orji M. G. Olaniyi K. Adeyemo T. (2022). Assessing micro, small and medium enterprises as an instrument for human capital development and poverty reduction in Abuja area councils of Nigeria. Br. Int. Hum. Soc. Sci. J. 4, 194–205. 10.33258/biohs.v4i2.649

[B68] Ortiz-Crespo B. Steinke J. Quirós C. F. van de Gevel J. Daudi H. Gaspar Mgimiloko M. van Etten J. (2021). User-centred design of a digital advisory service: enhancing public agricultural extension for sustainable intensification in Tanzania. Int. J. Agric. Sustain. 19, 566–582. 10.1080/14735903.2020.1720474

[B69] Palmén R. Arroyo L. Müller J. Reidl S. Caprile M. Unger M. (2020). Integrating the gender dimension in teaching, research content and knowledge and technology transfer: validating the EFFORTI evaluation framework through three case studies in Europe. Eval. Program Plan. 79, 101751. 10.1016/j.evalprogplan.2019.101751 31786403

[B70] Pan C. Abbas J. Álvarez-Otero S. Khan H. Cai C. (2022). Interplay between corporate social responsibility and organizational green culture and their role in employees' responsible behavior towards the environment and society. J. Clean. Prod. 366, 132878. 10.1016/j.jclepro.2022.132878

[B71] Parnell D. Widdop P. Bond A. Wilson R. (2020). COVID-19, networks and sport. Manag Sport Leisure 1–7. 10.1080/23750472.2020.1750100

[B72] Perveen S. Akram M. Nasar A. Arshad-Ayaz A. Naseem A. (2022). Vaccination-hesitancy and vaccination-inequality as challenges in Pakistan's COVID-19 response. J. Commun Psychol. 50, 666–683. 10.1002/jcop.22652 34217150 PMC8426931

[B73] Prideaux B. Thompson M. Pabel A. (2020). Lessons from COVID-19 can prepare global tourism for the economic transformation needed to combat climate change. Tourism Geograph. 22, 667–678. 10.1080/14616688.2020.1762117

[B74] Pu G. Qamruzzaman M. Mehta A. M. Naqvi F. N. Karim S. (2021). Innovative finance, technological adaptation and SMEs sustainability: the mediating role of government support during COVID-19 pandemic. Sustainability 13, 9218. 10.3390/su13169218

[B75] Rahmat A. Izudin A. (2018). Impact evaluation of community empowerment programs with the farmer managed extension. Eur. Res. Stud. 21, 225–35. 10.35808/ersj/997

[B76] Ratnawati K. (2020). The influence of financial inclusion on MSMEs' performance through financial intermediation and access to capital. J. Asian Financ. Econ. Bus. 7, 205–218. 10.13106/jafeb.2020.vol7.no11.205

[B77] Ratten V. (2020). Coronavirus (covid-19) and entrepreneurship: Changing life and work landscape. J. Small Bus Entrep. 32, 503–516 10.1080/08276331.2020.1790167

[B78] Ripain N. Amirul S. M. Mail R. (2017). Financial literacy and SMEs' potential entrepreneurs: the case of Malaysia. J. Admin. Bus. Stud. 3, 60–68. 10.20474/jabs-3.2.1

[B79] Salikin N. Ab Wahab N. Muhammad I. (2014). Strengths and weaknesses among Malaysian SMEs: financial management perspectives. Proc. Soc. Behav. Sci. 129, 334–340. 10.1016/j.sbspro.2014.03.685

[B80] Santos S. C. Neumeyer X. Morris M. H. (2019). Entrepreneurship education in a poverty context: an empowerment perspective. J. Small Bus. Manag. 57, 6–32. 10.1111/jsbm.12485

[B81] Sariwulan T. Suparno S. Disman D. Ahman E. Suwatno S. (2020). Entrepreneurial performance: the role of literacy and skills. J. Asian Financ. Econ. Bus. 7, 269–280. 10.13106/jafeb.2020.vol7.no11.269

[B82] Sarkar P. Kumar J. (2011). Impact of MGNREGA on reducing rural poverty and improving socio-economic status of rural poor: a study in Burdwan district of West Bengal. Agric. Econ. Res. Rev. 24, 437–448. 10.22004/ag.econ.119395

[B83] Schwert G. W. (2021). The remarkable growth in financial economics, 1974-2020. J. Finan. Econom. 140, 1008–1046. 10.1016/j.jfineco.2021.03.010

[B84] Setiawan R. Cavaliere L. P. L. Sankaran D. Rani K. M. Yapanto L. Laskar N. H. Christabel G. (2020). Access to Financial Services and Women Empowerment, through Microfinance eligibility (Doctoral dissertation, Petra Christian University).

[B85] Sharif S. P. Naghavi N. (2020). Family financial socialization, financial information seeking behavior and financial literacy among youth. Asia Pac. J. Bus. Admin. 12, 163–181. 10.1108/APJBA-09-2019-0196

[B86] Siekei J. Wagoki J. Kalio A. (2013). An assessment of the role of financial literacy on performance of small and micro enterprises: case of equity group foundation training program on SMEs in Njoro District, Kenya. Bus. Appl. Sci. 1, 250–271.

[B87] Singla A. Mallik G. (2021). Determinants of financial literacy: Empirical evidence from micro and small enterprises in India. Asia Pac. Manag. Rev. 26, 248–255. 10.1016/j.apmrv.2021.03.001

[B88] Siphesihle Q. Lelethu M. (2020). Factors affecting subsistence farming in rural areas of nyandeni local municipality in the Eastern Cape Province. South Afr. J. Agric. Exten. 48, 92–105. 10.17159/2413-3221/2020/v48n2a5402020

[B89] Sofo F. Wicks A. (2017). An occupational perspective of poverty and poverty reduction. J. Occup. Sci. 24, 244–249. 10.1080/14427591.2017.1314223

[B90] Sokoto A. A. Abdullahi Y. Z. (2013). Strengthening small and medium enterprises (SMEs) as a strategy for poverty reduction in North Western Nigeria. Am. J. Humanit. Soc. Sci. 1, 189–201. 10.11634/232907811301338

[B91] Sriary B. D. A. Nyoman Y. N. (2020). Strategy for improving the performance of MSMES through access to financial institutions. Eurasia Econ. Bus. 12, 17–28. 10.18551/econeurasia.2020-12

[B92] Srithongrung A. Yusuf J. E. W. Kriz K. A. (2021). A systematic public capital management and budgeting process, in Research Anthology on Preparing School Administrators to Lead Quality Education Programs (Hershey, PA: IGI Global). pp. 598–619. 10.4018/978-1-7998-3438-0.ch027

[B93] Stubbs T. Kring W. Laskaridis C. Kentikelenis A. Gallagher K. (2021). Whatever it takes? The global financial safety net, Covid-19, and developing countries. World Dev. 137, 105171. 10.1016/j.worlddev.2020.105171 32905064 PMC7462525

[B94] Tasos S. Amjad M. I. Awan M. S. Waqas M. (2020). Poverty alleviation and microfinance for the economy of Pakistan: A case study of Khushhali Bank in Sargodha. Economies. 8, 63. 10.3390/economies8030063

[B95] Tisdell C. Ahmad S. Agha N. Steen J. Verreynne M. L. (2020). Microfinance for wives: Fresh insights obtained from a study of poor rural women in Pakistan. J. Res. Gender Stud. 10, 9. 10.22381/JRGS10120201

[B96] Ullah A. Pinglu C. Ullah S. Abbas H. S. M. Khan S. (2021). The role of e-governance in combating COVID-19 and promoting sustainable development: a comparative study of China and Pakistan. Chin. Polit. Sci. Rev. 6, 86–118. 10.1007/s41111-020-00167-w PMC7644414 40477209

[B97] Ursachi G. Horodnic I. A. Zait A. (2015). How reliable are measurement scales? External factors with indirect influence on reliability estimators. Proc. Econ. Financ. 20, 679–686. 10.1016/S2212-567100123-9

[B98] Van der Sluis J. Van Praag M. Vijverberg W. (2008). Education and entrepreneurship selection and performance: A review of the empirical literature. J. Econom. Survey. 22, 795–841. 10.1111/j.1467-6419.2008.00550.x

[B99] Vermesan O. Friess P. (Eds.) (2022). Digitising the Industry Internet of Things Connecting the Physical, Digital and VirtualWorlds. Boca Raton, FL: CRC Press.

[B100] Villanueva A. Zhu Z. Liu Z. Peppler K. Redick T. Ramani K. (2020). Meta-AR-app: an Authoring Platform for Collaborative Augmented Reality in STEM Classrooms, in Proceedings of the 2020 CHI Conference on Human Factors in Computing Systems. pp. 1–14. 10.1145/3313831.3376146

[B101] Warue B. N. Wanjira T. V. (2013). Assessing budgeting process in small and medium enterprises in Nairobi's central business district: a case of hospitality industry. Int. J. Inf. Technol. Bus. Manag. 17, 1–11

[B102] Weiss H. B. Lopez M. E. Rosenberg H. (2010). Beyond Random Acts: Family, School, and Community Engagement as an Integral Part of Education Reform. National Policy Forum for Family, School, and Community Engagement. Harvard Family Research Project.

[B103] Williams T. A. Gruber D. A. Sutcliffe K. M. Shepherd D. A. Zhao E. Y. (2017). Organizational response to adversity: fusing crisis management and resilience research streams. Acad. Manag. Ann. 11, 733–769 10.5465/annals.2015.0134

[B104] Yamagishi K. Gantalao C. Ocampo L. (2021). The future of farm tourism in the Philippines: challenges, strategies and insights. J. Tour. Futur. 10.1108/JTF-06-2020-0101

[B105] Yasa Kerti N. N. Abdullah J. Sukaatmadja P. G. Kembar S. Marhaeni A. A. N. (2013). SME performance improvement and its effect on the poverty reduction in Bali. International Jurnal of Business Management Invention, 2(4), pp. 01–12.

[B106] Yulo Loyzaga A. Uy N. Lo D. Porio E. (2022). Private sector and higher educational institution partnerships to enhance resilience in the philippines: the experience of the national resilience council, in Safety and Resilience of Higher Educational Institutions. (Springer: Singapore). pp. 233–253. 10.1007/978-981-19-1193-4_14

