# Supplementary material for: Community extension MSME's entrepreneurial activities in relation to poverty reduction
Source: Front Sociol. 2022 Nov 28;7:1038006. doi: 10.3389/fsoc.2022.1038006 (PMC9742551; doi:10.3389/fsoc.2022.1038006)
Supplement: Supplementary file 1 [file Table_1.docx]

Appendix

| **Constructs/Indicators** | **Code** | **Items** | **References** |
| --- | --- | --- | --- |
| Access to Credit Facilities | ACF1 | Access to credit facilities have improved the level of their income | Kurgat, F., Owembi, K. O., & Omwono, G. A. (2017) |
|  | ACF2 | Access to credit facilities have enabled the family to meet their daily basic needs |  |
|  | ACF3 | Access to credit facilities have enabled the family to save more |  |
|  | ACF4 | Access to credit facilities have led to investments |  |
|  | ACF5 | Access to credit facilities empowered them to own property |  |
|  | ACF6 | Their life has improved after accessing credit facilities |  |
|  | ACF7 | The level of wealth has increased after accessing credit |  |
|  | ACF8 | There has been access to new technological tools and equipment that have improved our standard of living |  |
| Budgeting Financial Literacy | BFL1 | Acquired skills in profit planning, business financing planning and cash flow management | Siekei, J., Wagoki, J., & Kalio, A. (2013) |
|  | BFL2 | Budgeting skills acquired however played a key role in enhancing my entrepreneurship performance |  |
|  | BFL3 | Budgeting skills assisted me in augmenting my sales |  |
|  | BFL4 | Budgeting skills assisted me in growing my business profitability by providing a basis of establishing performance targets |  |
|  | BFL5 | Bookkeeping skills acquired through the skills training program |  |
|  | BFL6 | All shared skills are acquired |  |
|  | BFL7 | Budgeting skills were highly emphasized in all financial literacy training |  |
|  | BFL8 | The level of my budgeting skills is relevant to by business venture |  |
| Entrepreneurial Education | EE1 | helps the beneficiaries to seek for better business opportunities | Mwiya, B. M. K. (2014) |
|  | EE2 | prepares the beneficiaries against unemployment challenges |  |
|  | EE3 | contributes to the growth of their entrepreneurial skills |  |
|  | EE4 | helps the beneficiaries in enhancing my knowledge |  |
|  | EE5 | helps the beneficiaries in augmenting my income |  |
|  | EE6 | empowers the beneficiaries to strive a better standard of living |  |
|  | EE7 | educates the beneficiaries the value of entrepreneurship |  |
|  | EE8 | contributes to Increase the beneficiaries' understanding of the action I have to take during the business start-up |  |
|  | EE9 | enhances the beneficiaries' practical management skills to start a business |  |
|  | EE10 | enhances the beneficiaries' ability to develop networks |  |
|  | EE11 | enhances the beneficiaries' ability to identify an opportunity |  |
|  | EE12 | increase the beneficiaries' understanding of the attitudes, values and motivation of an entrepreneur |  |
|  | EE13 | prepares the beneficiaries to start a viable business |  |
| Entrepreneurial Performance | EP1 | Expansion of the beneficiaries' business is due to application of transferred technology. | Sariwulan, T., Suparno, S., Disman, D., Ahman, E., & Suwatno, S. (2020) |
|  | EP2 | Entrepreneurial performance is necessary for assessment the beneficiaries' business |  |
|  | EP3 | An increase in the number of entrepreneurs is an indication of an expansion of the business sector |  |
|  | EP4 | An increase in sales of products leads to growth and expansion of the beneficiaries' business |  |
|  | EP5 | Entrepreneurial performance encourages start-ups |  |
|  | EP6 | New businesses will emerge when beneficiaries practice entrepreneurship |  |
|  | EP7 | A good environment for entrepreneurship will encourage set-ups |  |
|  | EP8 | Business start-ups are essential for growth in productivity |  |
|  | EP9 | Entrepreneurial performance lays the ground for competitiveness |  |
|  | EP10 | Competition improves the quality of products |  |
| Poverty Reduction | PR1 | Food scarcity has reduced | Duclos, J. Y., & Tiberti, L., 2016; Dhongde, S., & Haveman, R., 2015 |
|  | PR2 | The family is enjoying the improved living standards |  |
|  | PR3 | The family can spare budget for clothing |  |
|  | PR4 | The family can spare budget for house repair |  |
|  | PR5 | The family can now afford bigger budget for medicine/health check-ups |  |
|  | PR6 | There is a consistent supply of clean water for the family |  |
|  | PR7 | The family can now buy additional appliances |  |
|  | PR8 | The family now has a budget for recreation |  |
|  | PR9 | The beneficiaries no longer have to work for many hours before purchasing necessities |  |
|  | PR10 | Daily income has increased to more than 275 pesos a day |  |
